# The *ndrg2* Gene Regulates Hair Cell Morphogenesis and Auditory Function during Zebrafish Development

**DOI:** 10.3390/ijms241210002

**Published:** 2023-06-11

**Authors:** Cheng Wang, Xin Wang, Hao Zheng, Jia Yao, Yuqing Xiang, Dong Liu

**Affiliations:** 1 Nantong Laboratory of Development and Diseases, School of Life Sciences, Co-Innovation Center of Neuroregeneration, Nantong University, Nantong 226001, China; chengwang@ntu.edu.cn (C.W.); 1909110229@stmail.ntu.edu.cn (J.Y.); xiangyq888@stmail.ntu.edu.cn (Y.X.); 2 Key Laboratory of Neuroregeneration of Jiangsu and Ministry of Education, Nantong University, Nantong 226001, China; ntuwx@ntu.edu.cn; 3 School of Medicine, Nantong University, Nantong 226001, China; 1931110169@stmail.ntu.edu.cn

**Keywords:** *ndrg2*, hair cell, hearing loss, zebrafish, Notch, cell differentiation

## Abstract

Damages of sensory hair cells (HCs) are mainly responsible for sensorineural hearing loss, however, its pathological mechanism is not yet fully understood due to the fact that many potential deafness genes remain unidentified. N-myc downstream-regulated gene 2 (*ndrg2*) is commonly regarded as a tumor suppressor and a cell stress-responsive gene extensively involved in cell proliferation, differentiation, apoptosis and invasion, while its roles in zebrafish HC morphogenesis and hearing remains unclear. Results of this study suggested that *ndrg2* was highly expressed in the HCs of the otic vesicle and neuromasts via in situ hybridization and single-cell RNA sequencing. *Ndrg2* loss-of-function larvae showed decreased crista HCs, shortened cilia, and reduced neuromasts and functional HCs, which could be rescued by the microinjection of *ndrg2* mRNA. Moreover, *ndrg2* deficiency induced attenuated startle response behaviors to sound vibration stimuli. Mechanistically, there were no detectable HC apoptosis and supporting cell changes in the *ndrg2* mutants, and HCs were capable of recovering by blocking the Notch signaling pathway, suggesting that *ndrg2* was implicated in HC differentiation mediated by Notch. Overall, our study demonstrates that *ndrg2* plays crucial roles in HC development and auditory sensory function utilizing the zebrafish model, which provides new insights into the identification of potential deafness genes and regulation mechanism of HC development.

## 1. Introduction

Hearing loss impairs the physical and mental health of patients that can be caused by a variety of factors, including genetic defects, ototoxic drugs, loud noise, and aging [1,2,3]. Sensory hair cells (HCs) are sensitive mechanoreceptors residing in the auditory organs that mediate the senses of hearing and balance and their damage is the most common cause of sensorineural hearing loss [1,2,3]. Due to the irreversibility and non-reproducibility of HC loss in mammalian inner ear, it is necessary to uncover the genes and pathways pivotal in HC development to provide the potential therapeutic treatment for hearing loss in humans. Hair cell development and survival undergoes the prosensory cell exiting cell cycle, expressing transcription factors, and terminally differentiating into sensory HCs and non-sensory supporting cells [4,5]. This extremely complicated and highly coordinated process is regulated by various key factors, such as *Atoh1* and *p27^Kip1^* and cell signaling pathways containing Notch/Wnt/Atoh1, MAPK, PI3K/Akt, calcium channels and oxidative stress/ROS [5,6,7,8]. Though a lot of efforts have been undertaken towards elucidating the molecular mechanisms of HC development and regeneration, there are many potential deafness genes that remain to be discovered and identified.

In auditory research, the model organisms of birds, chicks, zebrafish, mice, and rats are commonly used [4,9]. Mammalian animals have a typical structure of cochlea similar to humans, however, the complicated anatomy procedures and low throughput of the mammalian animal models bring about certain obstacles in application [9]. Zebrafish (*Danio rerio*), though devoid of cochlea, has auditory organs in the inner ear, namely the otic vesicle and the lateral line (LL) sensory system, which makes it suitable for using as an economic non-mammalian vertebrate model [10,11,12]. The otic vesicle, comprising the three pairs of semicircular canals, utricle and saccule, is responsible for balance and primarily hearing function [11,12]. Additionally, the LL system, that is exclusive in fish, detects sound and water flow, which is derived from primordium migration and deposition at stereotyped locations in the surficial body to form the mature neuromasts arrays [10,11,12]. Sensory HCs in zebrafish are tightly surrounded by supporting cells which is structurally and functionally similar to the mammalian inner ear. Combined with the innate characteristics of high fecundity, easy genetic manipulation and imaging in vivo, zebrafish has been widely used in drugs ototoxicity assessment, otoprotective agents screening, hearing-related gene functional identification and the mechanism of HC development and regeneration investigation [9,10,11,12,13,14].

N-myc downstream-regulated gene 2 (*NDRG2*) belonging to the *NDRG* gene family is extensively involved in the cellular biological processes including cell proliferation, differentiation, apoptosis, as well as cell migration and invasion [15,16,17]. Accumulated studies about the gene function suggest that *NDRG2*, also known as a tumor-suppressor gene, plays anti-proliferative and pro-apoptotic roles in many tumor issues, such as colorectal cancer, gastric cancer, hepatocellular carcinoma, etc. [17,18,19]. Meanwhile, NDRG2 protein level is negatively correlated with the tumor stage and aggressive behavior, which can act as a biomarker of tumor progression and prognosis [16,20]. In recent years, the pro-differentiation effect of the *NDRG2* gene has been reported in many types of cells and tissues [21,22,23,24,25,26,27]. For example, strong expression of the *NDRG2* gene was observed in the differentiation between monocyte and dendritic cells [21]. *NDRG2* could also promote the BMP2-induced osteoblastic differentiation and calcification, as well as colorectal cancer differentiation [22,23,24]. Additionally, *NDRG2*, principally expressed in the astrocytes of the central nervous system, is regarded to be a crucial regulator during the occurrence and development of neurological diseases, ranging from glioma to stroke, neurodegenerative diseases, and psychiatric disorders [15]. The homologous *ndrg2* gene of zebrafish is specifically expressed in the otic vesicles at the early phase embryos [28]. Moreover, from the data of our previous study on single-cell RNA sequencing of HCs, we know that the *ndrg2* gene is highly enriched in the HCs of zebrafish (GSE221471) [29]. These data aid us to reasonably infer that *ndrg2* is involved in HC development and auditory system formation to a certain extent. Unfortunately, the extent of the *ndrg2* gene’s function in the development of auditory organs remains largely unknown.

In this paper, the role of *ndrg2* in HC development and function of auditory sensory organs in zebrafish was explored. The loss-of-function models of *ndrg2* were established and morphological and functional phenotypes of HCs were analyzed. This work will not only deepen the understanding of *ndrg2* functions, but also give new insights into the regulation mechanism of HC development.

## 2. Results

### 2.1. The ndrg2 Gene Is Evolutionarily Conserved and Highly Expressed in the Otic Vesicle and Neuromasts of Zebrafish

**Figure 1:**
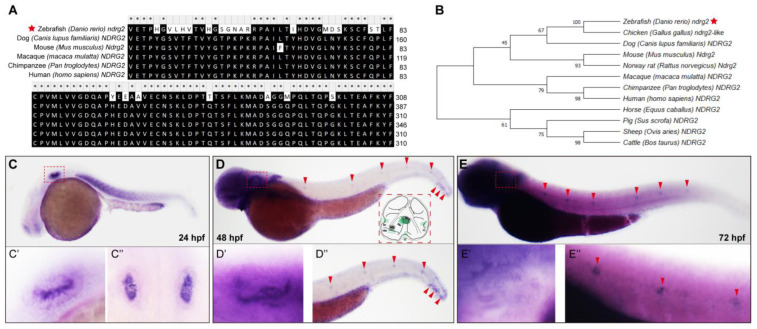
The *ndrg2* gene is conserved across multiple species and highly expressed in early developmental otic vesicles and neuromasts in posterior lateral line (pLL). (**A**) Amino acid sequence alignment of zebrafish (*Danio rerio*) *ndrg2* (marked with a red star), human *ndrg2* and homologous genes in several mammalian animals. (**B**) The established phylogenetic tree utilizing Neighbor-Joining method and MEGA software (version 6.0) containing zebrafish *ndrg2* (marked with a red star), human *ndrg2* and its orthologs in other species. (**C**–**E**) Expression profiles of *ndrg2* mRNA examined via whole-mount in situ hybridization (WISH) with the lateral view in zebrafish embryos at 24, 48 and 72 hpf, respectively. The positive signals of *ndrg2* mRNA were focused on the otic vesicle (marked with red dashed box) and neuromasts in pLL (marked with red arrowheads) during early embryo development. A schematic of the inner ear structure in zebrafish was shown in (**D**). UO, utricular otolith; SO, saccular otolith; UM, utricular macula; SM, saccular macula; AC, anterior crista; LC, lateral crista; PC, posterior crista. (**C’**,**D’**,**E’**) Magnified images of the positive signals in otic vesicle with lateral view in zebrafish at 24, 48 and 72 hpf, respectively. (**C’’**) A dorsal view of otic vesicles in zebrafish at 24 hpf. (**D’’**,**E’’**) Enlarged images of the positive signals in neuromasts in pLL (marked with red arrowheads) with lateral view in zebrafish at 48 and 72 hpf, respectively.

It has been reported that members of the *ndrg* family wildly exists in the metazoan across multiple species [18,30]. Zebrafish *ndrg2* gene (Gene ID: 494050; zgc: 101847) comprises 16 exons and encodes 368 amino acids. To characterize the evolutionary features of the *ndrg2* gene in detail, we analyzed amino acid sequences of the zebrafish *ndrg2* gene and its orthologs in human and also in other mammalian animals and constructed the phylogenetic tree using the Neighbor-Joining (NJ) method. The alignment results and the phylogenetic tree showed that the *ndrg2* gene was highly conserved in the lower vertebrates of mammals with a higher sequence homology in different species (Figure 1A,B).

To ascertain the temporal and spatial expression patterns of *ndrg2*, we traced the developmental process of zebrafish via the whole-mount in situ hybridization (WISH) using digoxigenin-labeled *ndrg2* mRNA antisense probe. At 24 hpf, the *ndrg2* gene was dramatically expressed in the otic vesicle (Figure 1C, red dashed box) and enlarged details were delineated both with lateral view and dorsal view (Figure 1(C’,C’’)). At 48 and 72 hpf, positive signals of *ndrg2* mRNA were detected not only in the otic vesicle (Figure 1D,E, red dashed boxes) but also in the neuromasts in the posterior lateral line (pLL) (Figure 1D,E, red arrowheads). The magnified graphs displayed that the high expression of *ndrg2* was restricted to the domains of HCs of cristae, maculae and neuromasts (Figure 1(D’,D’’,E’,E’’)). Additionally, the expression of *ndrg2* was also examined in the myotome, forebrain and central nervous system to a certain degree during embryonic development (Figure 1C–E). Further analysis of HCs single-cell RNA sequencing data in the previous work illustrated that the *ndrg2* gene was highly enriched in the clusters of crista HC, macula HC and neuromast HC as well as in the retinal ganglion cell and neuron, which was in agreement with the results of WISH (Figure S1). The high expression of *ndrg2* in the otic vesicle and the neuromasts in the pLL suggested that *ndrg2* was probably involved in the development of auditory organs in zebrafish.

### 2.2. Knockdown of the ndrg2 Gene Induces HC Defects Both in Ampulla Crista and pLL System

**Figure 2:**
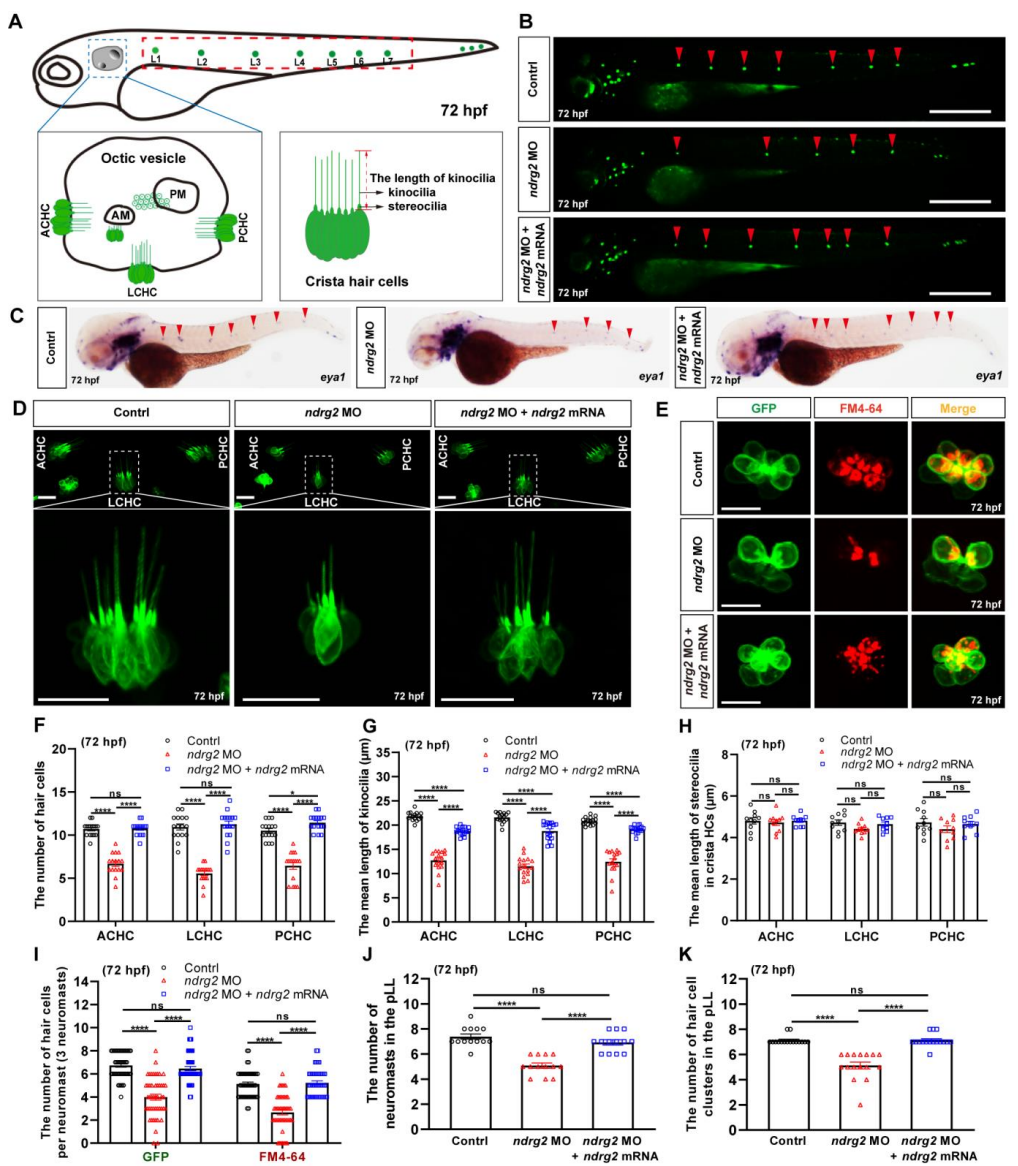
Knockdown of the *ndrg2* gene led to significantly reduced crista hair cells (HCs), shortened kinocilia, decreased neuromasts as well as reductive functional HCs. (**A**) Schematic structure of main auditory organs in zebrafish (*Danio rerio*) at 72 hpf, including otic vesicle and posterior lateral line (pLL) system. Detailed structures of both otic vesicle and crista HCs cluster were depicted and the typical three clusters of crista HCs comprised anterior crista hair cell (ACHC), lateral crista hair cell (LCHC) and posterior crista hair cell (PCHC). The neuromasts in pLL that were analyzed in this study were marked with red dashed box. (**B**) Representative fluorescence images of HC clusters (marked with red arrowheads) in pLL at 72 hpf in the control, *ndrg2* morphants, and *ndrg2* mRNA rescued groups, respectively. Scale bars: 500 µm. (**C**) Lateral views of *eya1* mRNA expression detected via whole-mount in situ hybridization (WISH) in normal, *ndrg2* morphants, and *ndrg2* mRNA rescued larvae at 72 hpf. The positive signals in neuromasts in the pLL were pointed with red arrowheads. (**D**) Representative fluorescence images of the three clusters of crista HCs at 72 hpf in control, *ndrg2* morphants, and *ndrg2* mRNA rescued groups, respectively. The details of LCHC (marked with white dashed box) were enlarged in corresponding groups. Scale bars: 20 µm. (**E**) Representative enlarged micrographs of HC cluster (green color) and functional HC cluster (red color) in single neuromast in pLL at 72 hpf in control, *ndrg2* morphants, and *ndrg2* mRNA rescued groups, respectively. Scale bars: 10 µm. (**F**–**H**) Statistical analysis of the number of crista HCs, the mean length of kinocilia and stereocilia at 72 hpf in the control, *ndrg2* morphants, and *ndrg2* mRNA rescued groups, respectively ((**F**,**G**) *n* = 16; (**H**), *n* = 10). (**I**) Statistical analysis of the number of HCs and functional HCs per neuromast at 72 hpf in control, *ndrg2* morphants, and *ndrg2* mRNA rescued groups, respectively (*n* = 48). (**J**,**K**) Statistical analysis of the number of HC clusters and neuromasts in pLL in control, *ndrg2* morphants, and *ndrg2* mRNA rescued groups, respectively ((**I**), *n* = 16; (**J**), *n* = 13). Symbols of * and **** above bars represent *p* < 0.05 and *p* < 0.0001, respectively. ns, no significance.

To explore the role of *ndrg2* in the development of auditory organ in zebrafish, *ndrg2* morphants were established via the microinjection of *ndrg2*-specific splicing-blocking morpholino based on the transgenic line *Tg(Brn3c:mGFP)*. The efficiency of morpholino microinjection was examined via PCR and results of multiple electrophoretic bands indicated that effective mis-splicing successfully occurred at the target site (Figure S2). Considering that the restrictive expression of *ndrg2* was mainly in the HC domains of the otic vesicle and the neuromasts in the pLL, we first observed and recorded the three typical clusters of crista HC in the otic vesicle (Figure 2A). Fluorescence images displayed remarkably reduced crista HCs in the anterior, lateral, and posterior locations and showed shortened kinocilia in the *ndrg2* morphants at 72 hpf (Figure 2D). A similar phenotype was detected in the *ndrg2* morphants at 96 hpf (Figure S3A), suggesting that defects of crista HCs were due to the specific knockdown of *ndrg2* rather than a developmental delay. Meanwhile, statistical results showed that the number of crista HCs and the mean length of kinocilia were both significantly decreased, while there was no significant difference in the mean length of stereocilia in the *ndrg2* morphants at 72 and 96 hpf, which was also well supported in the observed phenotype (Figure 2F–H and Figure S3D–F). Further studies found that there were no detectable morphological changes in both the utricular and saccular otoliths, as well as the utricular macula HCs in the otic vesicle during *ndrg2* knockdown (Figure S4).

To investigate the effect of *ndrg2* on the pLL system, *eya1* mRNA probe and GFP were selected to specifically recognize the neuromasts and HC clusters, respectively. Compared to the normal larvae, the *ndrg2* morphants at different developmental stages exhibited fewer neuromasts and HC clusters in the pLL (Figure 2B,C and Figure S3B). The consistent statistical results of the number of neuromasts and HC clusters in the pLL were also shown (Figure 2I,J and Figure S3G). To further describe the influence of *ndrg2* on the HCs in neuromasts, the functional HCs were labeled with FM4-64 vital dye that could rapidly enter the mature HCs through the functional mechanotransduction channels [31]. Representative images and statistical results indicated that both the number of HCs and functional HCs in single neuromasts in the pLL declined in the *ndrg2* morphants at different stages (Figure 2E,I and Figure S3C,H). Moreover, the defective phenotypes of HCs occurring in the *ndrg2* morphant could be partially rescued by microinjection of the exogenous *ndrg2* mRNA (Figure 2 and Figure S3). Altogether, knockdown of the *ndrg2* gene could specifically induce the abnormal HC morphogenesis of the otic vesicle and the pLL system, including decreased crista HCs, shortened kinocilia, reduced neuromasts and functional HCs.

### 2.3. Knockdown of the ndrg2 Gene Causes Weakened Response Behavior to Sound Vibration Stimuli with No Detectable Difference in Vestibulo-Ocular Reflex (VOR)

As known, sensory HCs are crucial mechanoreceptors in auditory organs that can convert external vibrational stimuli to electrophysiological signals to sense sound and balance [1,2,3]. Loss of sensory HCs commonly contributes to hearing loss and vestibular dysfunction [1,2,3]. Knockdown of *ndrg2* leads to defective phenotypes of HCs; whether that knockdown influences the auditory and balance function of zebrafish remains to be verified. Here, a customized vestibulo-ocular reflex (VOR) testing was performed to examine the linear VOR in zebrafish larvae at 5 dpf, evoked by the head motion to the earth horizontal axis (Figure S5A). Statistical results showed that there was no significant difference in the amplitude of eye movements in the larvae with *ndrg2* deficiency (Figure S5B), which indicated that knockdown of *ndrg2* could not induce the changes in linear VOR at 5 dpf.

**Figure 3:**
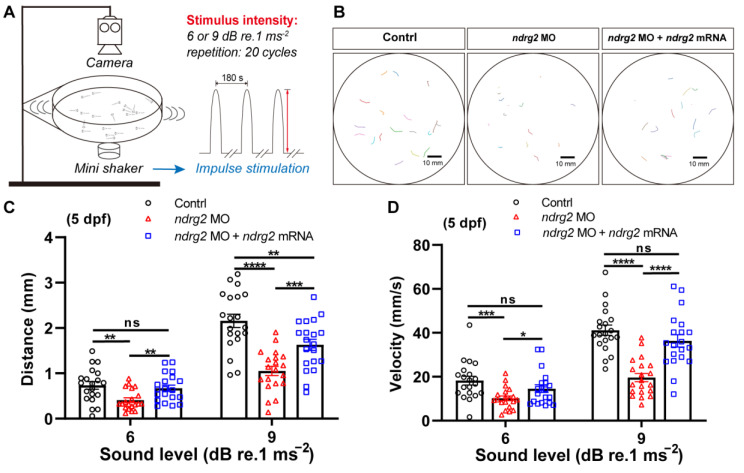
Knockdown of the *ndrg2* gene induced weakened response behavior to sound stimuli in zebrafish (*Danio rerio*) at 5 dpf. (**A**) Schematic diagram of the devices used in startle response assay and sound vibration stimuli modes applied to larvae. (**B**) Extracted locomotion trajectories from larvae with C-shape motion under one-time stimulus of 9 dB re.1 ms^−2^ sound level with 180 Hz tone bursts in control, *ndrg2* morphants, and *ndrg2* mRNA rescued groups, respectively. (**C**,**D**) Statistical analysis of the mean distance and peak velocity of locomotion under two levels stimuli in normal, *ndrg2* morphants, and *ndrg2* mRNA rescued larvae at 5 dpf, respectively (*n* = 20). Symbols of *, **, ***, and **** above bars represent *p* < 0.05, *p* < 0.01, *p* < 0.001, and *p* < 0.0001, respectively. ns, no significance.

Then, a startle response test was conducted to further explore the functional role of *ndrg2* in the hearing ability of zebrafish. Two magnitudes of sound-evoked stimuli with 180 Hz tone bursts were applied and the larvae with C-shaped motion after each stimulus were calculated and analyzed (Figure 3A,B). Results showed that the response behavior of larvae was stimuli magnitude-dependent while the *ndrg2* morphants appeared to have more insensitive responses to sound vibration stimuli compared to the wild-type larvae at 5 dpf, which was reflected in the reductive mean distance and the peak velocity of movement (Figure 3C,D). Furthermore, injection of *ndrg2* mRNA in vitro, to a degree, could remedy the slow response behavior of larvae to sound vibration stimuli caused by the deficiency of *ndrg2* (Figure 3C,D). It implied that knockdown of the *ndrg2* gene could impair the function of auditory organs in zebrafish larvae.

### 2.4. Knockout of the ndrg2 Gene Leads to HC Development Disorder and Sensory Dysfunction to Sound Vibration Stimuli

**Figure 4:**
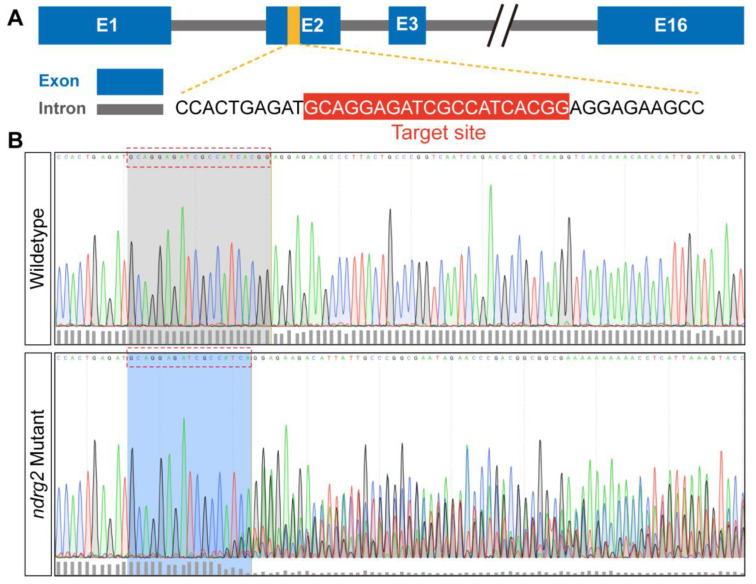
CRISPR/Cas9-mediated *ndrg2* mutation was successfully generated at the target site. (**A**) Schematic diagram of zebrafish (*Danio rerio*) *ndrg2* genomic structure and *ndrg2*-specific sgRNA targeting exon 2 in the CRISPR/Cas9 system. (**B**) Multiple mutations occurred at the target site in *ndrg2* mutants in comparison with wild-type zebrafish.

To further demonstrate the developmental and functional roles of *ndrg2*, first, the *ndrg2* knockout mutants were generated based on *Tg(Brn3c:mGFP)* transgenic zebrafish using the CRISPR/Cas9 genome-editing strategy. A sgRNA targeting the exon 2 of the *ndrg2* gene was designed and co-injected with Cas9 mRNA into one-cell stage embryos for CRISPR/Cas9-mediated editing of *ndrg2* (Figure 4A). The effectiveness of the *ndrg2* knockout was identified by sequencing an approximately 410 bp genomic DNA fragment containing the target site. Results showed that multiple peaks curve appeared behind the target site in the *ndrg2* mutant in comparison with the wild-type larvae, indicating that the mutation targeting exon 2 has successfully occurred (Figure 4B).

**Figure 5:**
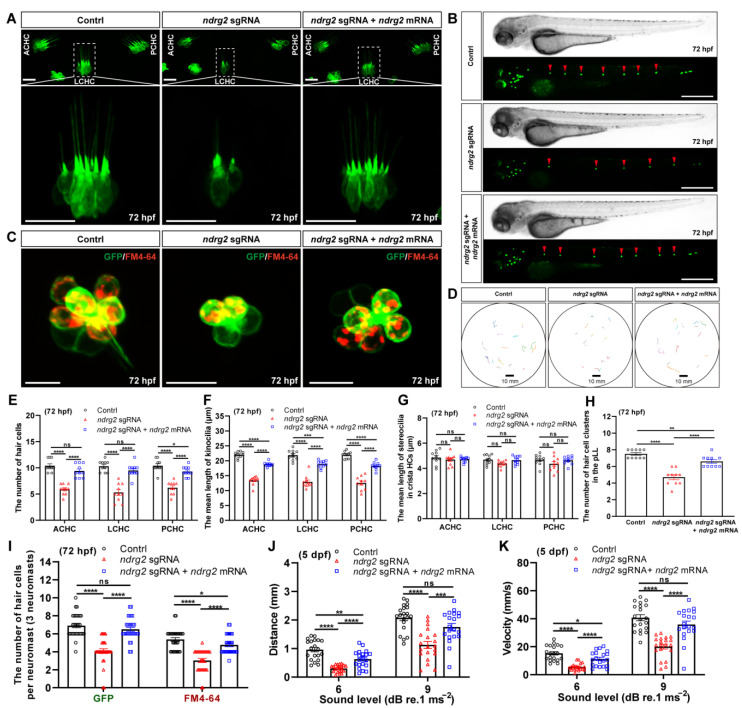
Knockout of the *ndrg2* gene disrupted hair cell (HC) morphogenesis and caused defective sensory ability to sound vibration stimuli in zebrafish (*Danio rerio*). (**A**) Representative fluorescence graphs of the typical three clusters of crista HCs in normal, *ndrg2* mutants, and *ndrg2* mRNA rescued larvae at 72 hpf, respectively. The details of lateral crista hair cell (LCHC) (marked with white dashed box) were magnified in corresponding larvae. Scale bars: 20 µm. (**B**) Phase-contrast and fluorescence images of HC clusters (marked with red arrowheads) in posterior lateral line (pLL) at 72 hpf from the control, *ndrg2* mutants, and *ndrg2* mRNA rescued groups, respectively. Scale bars: 500 µm. (**C**) Overlapped fluorescence images of a representative neuromast in pLL from normal, *ndrg2* mutants, and *ndrg2* mRNA rescued larvae at 72 hpf, respectively. The green and red signals represented HCs and functional HCs, respectively. Scale bars: 10 µm. (**D**) Swimming trajectories extracted from once startle response behavior to sound stimuli of 9 dB re.1 ms^−2^ with 180 Hz tone bursts in the normal, *ndrg2* mutants, and *ndrg2* mRNA rescued larvae at 5 dpf, respectively. (**E**–**I**) Statistical analysis of the morphological phenotypes of HCs emerging in ampulla crista of otic vesicle and neuromasts in pLL at 72 hpf in the control, *ndrg2* mutants, and *ndrg2* mRNA rescued groups, respectively ((**E**–**H**), *n* = 10; (**I**), *n* = 30). (**J**,**K**) Statistical analysis of the mean distance and peak velocity of locomotion under two levels stimuli in the control, *ndrg2* mutants, and *ndrg2* mRNA rescued groups, respectively (*n* = 20). Symbols of *, **, ***, and **** above bars represent *p* < 0.05, *p* < 0.01, *p* < 0.001, and *p* < 0.0001, respectively. ns, no significance.

To determine the effect of *ndrg2* knockout on HCs morphogenesis, the developmental processes of HCs in ampulla crista and neuromasts in the pLL were monitored and analyzed. Results showed that similar to the phenotype of the *ndrg2* morphants, the three typical clusters of crista HCs were reduced and it was accompanied by the shortened kinocilia and unchangeable stereocilia in the *ndrg2* mutants at 72 hpf (Figure 5A). Meanwhile, significantly reduced HC clusters in the pLL were observed in the larvae at 72 hpf during *ndrg2* knockout (Figure 5B). Magnified graphs of single neuromast in the pLL illustrated that the larvae at 72 hpf that lacked *ndrg2* exhibited fewer HCs as well as functional HCs compared to the normal larvae (Figure 5C). The statistical results consistent with the above-observed phenotypes were also shown (Figure 5E–I). Similar changes in the HCs in ampulla crista and the pLL system were also detected in the *ndrg2* mutants at 96 hpf (Figure S6). Furthermore, startle response assays suggested that the *ndrg2* mutants were desensitized in response to external sound vibration stimuli with declined mean distance and peak velocity of movement (Figure 5D,J,K). In addition, morphological and functional defects of the HCs emerging in the *ndrg2* mutants could be partially recovered by microinjection of *ndrg2* mRNA (Figure 5 and Figure S6). The above results indicated that the *ndrg2* gene deeply influenced HC morphogenesis and the function of auditory organs in zebrafish.

### 2.5. Loss of the ndrg2 Gene Affects HC Differentiation Involved in Notch Signaling Pathway

**Figure 6:**
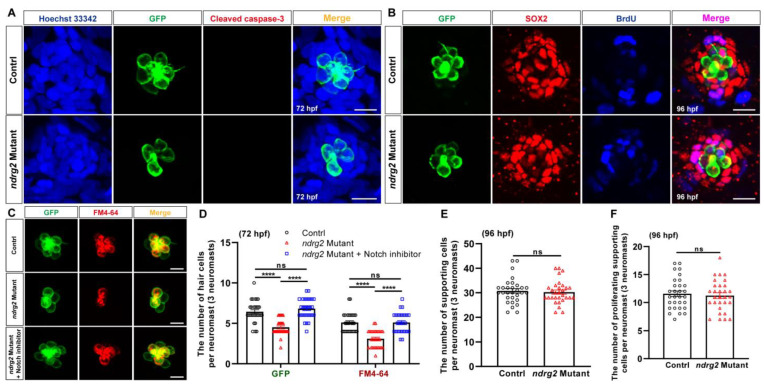
Loss of the *ndrg2* gene in *Tg(Brn3c:mGFP)* zebrafish (*Danio rerio*) influenced hair cells’ (HCs’) differentiation through Notch signaling without changes in HCs’ apoptosis and supporting cells. (**A**) Results of HC apoptosis assay in normal and *ndrg2* mutant larvae at 72 hpf. Immunofluorescence images were shown to recognize the nucleus (blue color), HC (green color), and cleaved caspase-3 signal (red color) in a single HC cluster in posterior lateral line (pLL). Scale bars: 10 µm. (**B**) Results of supporting cell proliferation assay in normal and *ndrg2* mutant larvae. Immunofluorescence micrographs were displayed to identify the HC (green color), supporting cell (red color) and proliferating cell (blue color) in a single neuromast in pLL at 96 hpf. Alexa Fluor^TM^ 647 used to detect BrdU was painted in pseudo blue color to distinguish from the red color of Alexa Fluor^TM^ 555 labeling SOX2. The purplish-red signals in the last column of overlapped fluorescence images represented the proliferating supporting cells. Scale bars: 10 µm. (**C**) Representative images of HC cluster (green color) and functional HC cluster (red color) in single neuromast in pLL at 72 hpf in control, *ndrg2* mutants and treatment with Notch inhibitor LY411575 groups, respectively. Scale bars: 10 µm. (**D**) Statistical analysis of the number of HCs and functional HCs per neuromast at 72 hpf in control, *ndrg2* mutants and treatment with Notch inhibitor LY411575 groups, respectively (*n* = 30). (**E**,**F**) Statistical analysis of the number of supporting cells and proliferating supporting cells per neuromast at 96 hpf in normal and *ndrg2* mutant larvae (*n* = 30). Symbols of **** above bars represent *p* < 0.0001. ns, no significance.

To further clarify the cell biological mechanism of HC loss induced by *ndrg2* deficiency, an antibody against cleaved caspase-3, an apoptosis marker was used to examine the cell apoptosis signals in the *ndrg2* mutants. Fluorescence images showed that there was no detectable signal of the cleaved caspase-3 co-localizing with HCs, both in the normal larvae and the *ndrg2* mutants, suggesting that cell apoptosis was not the cause of HC loss (Figure 6A). It is well known that HCs innately fail to autonomously proliferate and to regenerate via the mitosis and direct transdifferentiation of the supporting cells [3,4]. Whether the loss of the *ndrg2* gene disrupts the development of supporting cells serving as an important source of HCs remains to be elucidated. Here, an anti-SOX2 antibody was used to label the supporting cells and BrdU cell proliferation assay was conducted. Cells with both SOX2-positive and BrdU-positive signals represented the proliferating supporting cells. Immunofluorescence and statistics results illustrated that there was no significant difference in the number of supporting cells and proliferating supporting cells during *ndrg2* knockout (Figure 6B,E,F). It implied that the absence of *ndrg2* did not influence the proliferation and development of the supporting cells. Therefore, we speculated that the phenotype of HC loss emerging in the *ndrg2* mutants was due to the defects of HC differentiation. Notch-mediated lateral inhibition is widely believed to regulate HC versus supporting cell specification while suppression of Notch signaling promotes the differentiation of HCs from the supporting cells [4,6,32,33,34,35]. To verify whether the *ndrg2* gene affected HC differentiation through the Notch signaling pathway, a Notch γ-secretase inhibitor LY411575 was used to treat the *ndrg2* mutants. Representative graphs and statistical results showed that reduced HCs and functional HCs in a single neuromast in the pLL in the *ndrg2* mutants could be markedly recovered by blocking the Notch signaling pathway (Figure 6C,D). It revealed that *ndrg2* deficiency was closely related with HC differentiation mediated by the Notch signaling pathway.

## 3. Discussion

The N-myc downstream-regulated gene (*NDRG*) family is composed of four members, namely *NDRG1*, *NDRG2*, *NDRG3* and *NDRG4* [18,30,36]. The NDRG proteins are characterized by a conserved NDR domain including the alpha/beta (α/β) hydrolase motif but without enzymatic activity and have 57–65% identical amino acid sequences [18,30,36]. The *NDRGs* conservatively exist in metazoan across multiple species [18,30]. It was well verified in the phylogenetic tree of *NDRG2* constructed using the NJ method and the *ndrg2* gene of zebrafish shared more than 80% similarity with the human *NDRG2* (Figure 1A,B). The functions of *NDRG2* have been widely discussed and accumulative researches suggest its essential roles in a variety of cell biological processes. The *NDRG2* was considered as a stress-responsive gene in normal cells activated by cell stress stimuli, such as hypoxia, lower metabolic processes including inducing cell death, inhibiting cell proliferation and protein synthesis, etc. [18,30,36]. As a tumor suppressor, *NDRG2* exerted critical roles in anti-proliferation, pro-apoptosis as well as in restraining invasion and metastasis in different cancers [16,17,18,19,20]. It was found that NDRG2 as a marker protein for brain astrocytes was implicated in several neurological diseases [15]. In recent years, different studies reported that *NDRG2* was a differentiation-related gene in many types of cells and tissues [21,22,23,24,25,26,27,37,38,39]. Researchers indicated that *NDRG2* modulated the dendritic cell differentiation from monocytes and cytokine production, such as IL-10 [21,37]. Moreover, *NDRG2* promoted the expression of GATA-1 which induces the differentiation from megakaryocytes to erythrocytes [26]. Furthermore, *NDRG2* was capable of promoting BMP2-induced osteoblastic differentiation and inhibiting osteoclast differentiation, as well as controlling the vertebral specification in differentiating somites [23,27,38]. The *NDRG2* expression was higher in differentiated myotubes in comparison with undifferentiated myoblasts, highlighting its functions in myoblast growth and differentiation as well [25]. Additionally, *NDRG2* not only facilitates colorectal cancer differentiation, but also reduces the differentiation of macrophages into tumor-associated macrophages in the tumor microenvironment [22,24,39].

Zebrafish *ndrg* family consists of six paralogue members containing *ndrg1a, 1b, 2, 3a, 3b and 4*, which was expressed in metabolically demanding organs of zebrafish embryo, such as the brain, kidney, and heart [30]. Reports suggested that *ndrg2* gene was mainly restricted to express in the otic vesicles, brain, retina and somite during early embryonic development stages in zebrafish [28,30]. Similar expression patterns of *ndrg2* were also observed in our WISH results (Figure 1C–E). Nevertheless, the distinction was that positive signals of *ndrg2* were examined in neuromasts in the pLL in zebrafish embryos at 48 hpf and 72 hpf (Figure 1D,E). This finding was firmly confirmed by the results of HCs single cell sequence in our previous work which stated that *ndrg2* was highly enriched in neuromasts HCs of clusters 0 and 7 (Figure S1). A better overall and systematic expression profile of *ndrg2* was provided for insights into the potential tissue-specific functions. Here, the functions of *ndrg2* in tissues with specific expression have been extensively investigated, such as nervous system, retina, somite, etc. [15,27,40]. Unfortunately, the role of *ndrg2* in the auditory organs remains poorly understood. Considering the specific high expression of *ndrg2* both in the otic vesicles and neuromasts in the pLL, we speculated that the *ndrg2* gene probably participated in HCs’ development in zebrafish. To test this hypothesis, firstly, the *ndrg2* morphants and mutants were successfully constructed (Figure 4 and Figure S2). The defective phenotypes of HCs dramatically emerged both in ampulla crista and the pLL system, including reduced crista HCs, shortened kinocilia, decreased neuromasts as well as functional HCs, which could be partially restored by *ndrg2* mRNA rescued experiment (Figure 2, Figure 5 and Figures S3 and S6). Howerver, not all HCs in zebrafish would appear abnormality in morphology. For example, there was no detectable morphological difference in utricular macula HCs and otiliths in absence of *ndrg2* (Figure S4).

As we know, HCs act as important mechanoreceptors and are responsible for sensing sound, maintaining balance and gravity and its loss might lead to auditory and vestibular dysfunction in zebrafish. Therefore, the conventional behavior tests, VOR and startle response, were conducted with zebrafish larvae at 5 dpf to evaluate the influence of *ndrg2* on vestibular and auditory function, respectively (Figure 3, Figure 5 and Figure S5). It has been reported that VOR can be evoked by both linear and angular accelerations [41]. The otolith organs that sense linear acceleration and gravity matured after 5 dpf in zebrafish, whereas semicircular canals that sense angular acceleration of the rotation matured after 35 dpf [41,42]. The VOR testing system in this study was obtained from the Southern University of Science and Technology and designed to examine linear VOR in zebrafish larvae evoked by the changed intersection angle between gravity and otolith organ [43]. The linear VOR assay in this study mainly reflected the vestibular function associated with otolith organs. There was no detectable change in linear VOR in the *ndrg2* morphants, which was consistent with the observed normal phenotypes of otoliths and utricular macula HCs (Figures S4 and S5). Though crista HCs vertically inserted into the ampulla of semicircular canals exhibited defective phenotype in the *ndrg2* morphants (Figure 2 and Figure S3), their vestibular-related function could not be embodied and examined in the linear VOR assay with larvae at 5 dpf. The startle response is a behavior closely related to the auditory function of HCs and contains the typical processes through which the fish body bends into a characteristic “C” shape within 10 ms after acoustic stimuli followed by a small reversed curve and fast swimming [41]. The startle response can be triggered by sound stimuli from 5 dpf and throughout adulthood with similar intensity threshold and frequency range, which provides a reliable assessment of the hearing change [41,44]. The zebrafish lateral line can sense flows and low-frequency waves up to 200 Hz, while the inner ear can detect vibrations higher than 100 Hz [44,45]. In this study, the 180 Hz tone bursts with two different sound levels of 6 or 9 dB re.1 ms^−2^ were applied. The weakened startle response behaviors were observed in the *ndrg2* morphants and mutants (Figure 3 and Figure 5), reflecting the collaborative outcomes of the inner ear and lateral line to sound vibration stimuli. It was likely related with the decreased crista HCs and lessened neuromasts HCs in the pLL (Figure 2, Figure 5 and Figures S3 and S6). However, saccular macula, another important auditory sensory component of the inner ear, was not characterized in this work due to the imaging technical difficulties, which probably was an obstacles in fully realizing the relationship between morphological and functional changes of HCs. In general, the results of the startle response assay indicated that the *ndrg2* gene regulated the auditory sensory function in zebrafish. A publication reported that *ndrg2* was up-regulated in zebrafish when exposed to simulated gravity environment, implying the correlations of *ndrg2* with sensory behaviors [46].

Development of HCs is an extremely complex and highly coordinated process and it is regulated by a series of key factors involving multiple signaling pathways, such as Notch/Wnt/Atoh1, MAPK, PI3K/Akt, calcium channels and oxidative stress/ROS [4,5,6,7,8]. The classical and conserved Notch signaling pathway plays critical roles in the development and regeneration of HCs [3,4,5,6,32]. Studies suggested that Notch-mediated lateral inhibition determined the cell fate of the sensory epithelium and modulated the HCs versus supporting cell specification by activation of Notch signaling to inhibit the neighboring HCs from differentiating into the same cell type, and then to adopt the alternative fate, that is, supporting cells [4,6,32]. It was known that HCs were regenerated via mitosis or direct transdifferentiation of the supporting cells [3,4]. Researchers found that supporting cells were induced to transdifferentiate into HCs when Notch signaling was suppressed by the treatment of γ-secretase inhibitor [33,34,35]. Here, knockout of the *ndrg2* gene induced loss of HCs along with no detectable cell apoptosis signals of the cleaved caspase-3 (Figure 6A), indicating that HC apoptosis was not the causes of their decline. Meanwhile, supporting cells as an important source of HCs whose mitosis was not affected by the absence of *ndrg2*, reflected the non-significant changes in the number and morphology of the proliferating supporting cells (Figure 6B,E,F). Moreover, blocking Notch signaling with the γ-secretase inhibitor LY411575 helped in recovering the loss of HCs in the *ndrg2* mutants (Figure 6C,D), revealing that *ndrg2* was implicated in the Notch signaling pathway. A publication identified that *ndrg2* regulated the cortical neurogenesis at the lesion boundary after injury associated with the Notch signaling pathway [47]. A new research reported that *ndrg2* was involved in regeneration of impaired HCs in neuromasts via the Notch signaling pathway [48]. Building on these findings, the putative role of *ndrg2* was known that probably acted as an upstream negative regulator gene of the Notch signaling pathway to influence the differentiation of HCs. The *ndrg2* is likely a HC differentiation-related gene and its deficiency triggered Notch activation to inhibit differentiation of supporting cells into HCs, ultimately leading to the loss of HCs.

## 4. Materials and Methods

### 4.1. Zebrafish Lines and Maintenance

The transgenic zebrafish line *Tg(Brn3c:mGFP)* and wild-type AB strain were bred at 28.5 °C and maintained following the standard procedures as described in previous protocols [9]. Spawning embryos were raised with E3 medium in a constant temperature incubator at 28.5 °C. To avoid pigment formation, embryo medium was replaced with 0.2 mM PTU (1-phenyl-2-thiourea, Sigma, Saint Louis, MO, USA) solution at 20 h post-fertilization (hpf). The embryonic developmental stages were segmented according to the standard guidelines. All animal experiments were approved by the Animal Care and Use Committee of Nantong University. Permission No. S20210310-007, Approval date: 10 March 2021.

### 4.2. Whole-Mount In Situ Hybridization

The whole-mount in situ hybridization (WISH) of zebrafish was performed according to the following standard procedures. A 531 bp cDNA fragment of zebrafish *ndrg2* gene was amplified via PCR using designed primers (Table S1) and was cloned into the pGEM-T-easy vector. After linearization of the pGEM-T-easy inserting *ndrg2* fragment, the DIG RNA Labeling Kit (SP6 & T7) (Roche, #11175025910, Indianapolis, IL, USA) was used to prepare digoxigenin-labeled *ndrg2* antisense mRNA probes through transcription in vitro. Subsequently, embryos at different developmental stages were hybridized with *ndrg2* mRNA probe overnight after a series of treatment, including fixation in 4% (*w*/*v*) paraformaldehyde (PFA), digestion in proteinase K and incubation with a pre-hybridized mix. Finally, the alkaline phosphatase (AP)-conjugated antibody against digoxigenin (Roche, #11093274910) and the AP-substrate NBT/BCIP solution (Roche, #11681451001) were used to detect the *ndrg2* expression. To specifically recognize the neuromasts in the pLL, *eya1* mRNA probe was prepared as described above with designed primers (Table S1).

### 4.3. Morpholino Microinjection and mRNA Synthesis

For inhibiting the expression of *ndrg2*, *ndrg2*-specific splicing-blocking morpholinos was designed and procured from Gene Tools, Inc. and the precise sequence was (5′-ATC ATC TGA GAC TTA CTG TCC ATT C-3′). The powder of morpholino was dissolved and diluted in RNase-free water to obtain the work solution with a 0.3 mM final concentration for subsequent operations. About 2 nL dose of morpholino work solution was microinjected into zebrafish embryos at the one-cell stage. To examine the morpholino efficiency, embryos injected with morpholino were collected to extract RNA which then was subjected to reverse transcript cDNA. The designed primers franking on exon 1 and exon 8 (Table S1) were used to amplify the fragment containing the mis-splicing target site which was located at the connection of exon 4 and intron 4. For rescue experiments, exogenous *ndrg2* mRNA was first synthesized by transcription in vitro. Briefly, the designed primers, *ndrg2*-mRNA-BamHI-F and *ndrg2*-mRNA-XbaI-R (Table S1), were used to amplify an approximately 1400 bp DNA containing the entire *ndrg2* coding sequence. Then, the pCS2+ vector inserted into the amplified fragment was linearized as a template for transcription of *ndrg2* mRNA utilizing the SP6 mMESSAGE mMACHINE Kit (Invitrogen, #AM1340, Waltham, MA, USA). After being purified using the RNeasy Mini Kit (Qiagen, #74104, Hilden, Germany), *ndrg2* mRNA with a 50 ng/μL concentration was co-injected into one-cell stage embryos with morpholino or sgRNA for rescue experiments.

### 4.4. CRISPR/Cas9-Mediated Mutagenesis

The *ndrg2* mutant zebrafish were generated via CRISPR/Cas9-mediated gene editing technology. The single guide RNA (sgRNA) specifically targeting exon 2 of *ndrg2* was first synthesized as follows. A sgDNA was cloned via PCR with a forward primer containing the *ndrg2* gene-targeting site (5′-GCA GGA GAT CGC CAT CAC GG-3′) and a universal reverse primer (Table S1). Then, sgDNA as a template and MAXIscriptTM T7 Transcription Kit (Invitrogen, #AM1314) were occupied to obtain sgRNA via transcription in vitro. Meanwhile, Cas9 mRNA was synthesized in vitro using linearized pXT7-Cas9 plasmid and mMESSAGE mMACHINE^TM^ T7 Kit (Invitrogen, #AM1344). Subsequently, about 2 nL mixture of 300 ng/μL Cas9 mRNA and 100 ng/μL *ndrg2* sgRNA was microinjected into zebrafish embryos at one-cell stage. The *ndrg2* gene mutation in G0 embryos was examined via PCR using designed sequencing primers franking on intron 1 and intron 2 (Table S1) and the amplified fragments containing the targeting site were sequenced.

### 4.5. FM4-64 Staining, Inhibitor Treatment and Cell Apoptosis Assay

To recognize the functional HCs in neuromasts, FM4-64 staining experiment was conducted as follows. The free swimming larvae were incubated in 3 μM FM4-64 vital dye (ex/em = 558/734 nm, Molecular Probe, #T13320, Eugene, OR, USA) for 45 s at room temperature (RT) in the dark. Then, the larvae were imaged directly after removing the dye solution and gently rinsing with the embryo medium. To inhibit Notch signaling, the *ndrg2*-mutant larvae at 48 hpf were treated with the 50 μM γ-secretase inhibitor LY411575 (Selleckchem, #S2714, Houston, TX, USA) for 24 h for subsequent imaging. To detect caspase-3 activation signals in neuromasts, cell apoptosis assay was performed as follows. Briefly, the larvae at 72 hpf were fixed overnight in 4% PFA at 4 °C and then permeated with 1% Triton X-100 for 0.5 h at RT followed by blocking with 10% donkey serum for 1 h at 37 °C. Subsequently, the larvae were incubated with the primary antibodies of a chicken polyclonal anti-GFP (1:500 dilution, Abcam, #ab13970, Singapore) and a rabbit monoclonal anti-cleaved caspase-3 (1:500 dilution, CST, #9664, Singapore) overnight at 4 °C, which was detected via the secondary antibodies of a Alexa Fluor^TM^ 488 goat anti-chicken lgG (H + L) (1:1000 dilution, Invitrogen, #A-11039) and a Alexa Fluor^TM^ 555 donkey anti-rabbit lgG (H + L) (1:1000 dilution, Invitrogen, #A-31572), respectively. Finally, Hoechst 33342 (10 µg/mL, Invitrogen, #62249) dye was used to stain cell nuclei.

### 4.6. Supporting Cell Proliferation Assay

To characterize the proliferation of supporting cells, BrdU staining and immunofluorescence assays were conducted as follows. The larvae at 72 hpf were placed in 10 mM BrdU solution (Sigma-Aldrich, #B5002-5G, Saint Louis, MO, USA) for 24 h at 28.5 °C. After washing in embryo medium to remove the BrdU, the larvae at 96 hpf were fixed overnight in 4% PFA at 4°C and permeated with 1% Triton X-100 for 0.5 h at RT. Then, the larvae were immersed in 2 N HCl for 0.5 h at 37 °C followed by blocking with 10% donkey serum for 1 h at 37 °C. After that, the primary antibodies of a chicken polyclonal anti-GFP (1:500 dilution, Abcam, #ab13970), a rabbit polyclonal anti-SOX2 (1:500 dilution, Abcam, #ab97959) and a mouse monoclonal anti-BrdU (1:500 dilution, Santa Cruz Biotechnology, #sc-32323, Dallas, TX, USA) were used to label the HCs, supporting cells and proliferative cells, respectively. At last, the secondary antibodies of a Alexa Fluor^TM^ 488 goat anti-chicken lgG (H + L) (1:1000 dilution, Invitrogen, #A-11039), a Alexa Fluor^TM^ 555 donkey anti-rabbit lgG (H + L) (1:1000 dilution, Invitrogen, #A-31572) and a Alexa Fluor^TM^ 647 donkey anti-mouse lgG (H + L) (1:1000 dilution, Invitrogen, #A-31571) were used to detect the primary antibodies, respectively.

### 4.7. Vestibulo-Ocular Reflex (VOR) Assay

A customized VOR testing system was obtained from the Southern University of Science and Technology [43] and used to quantify linear VOR in zebrafish larva, evoked by the head motion to the earth horizontal axis. The detailed procedures for linear VOR testing are as follows. The zebrafish larva was gently mounted in the larva-shaped chamber in a dorsal-up position with the tail glued by 5% methylcellulose and covered with a piece of glass coverslip on the chamber. After adding E3 embryo media in the head region, the chamber unit was then mounted on a device for quantifying linear VOR. After aligning the larval eyes to the center of the infrared camera, the platform started to rotate back and forth around a horizontal axis at a speed of 30 rpm, and the VOR was recorded by the camera.

### 4.8. Startle Response Assay

The locomotion behavior of zebrafish was tested via the startle response assay and the detailed operation was performed as follows. A plastic plate attached to a mini vibrator was used to place 20 normal larvae at 5 days post-fertilization (dpf), while an infrared digital video tracking system was used to monitor their swimming behavior. The 180 Hz tone bursts with two different sound levels of 6 or 9 dB re.1 ms^−2^ were applied to the amplifier to drive the vibrator. The acoustic vibration stimuli lasting for 30 ms with a 180 s inter-stimulus interval were set and applied. Each sound vibration stimulus level were repeated for 20 times and the locomotion behavior of the larvae with C-shape motion to this stimulus was recorded. Finally, the movement typical parameters of mean distance and peak velocity were analyzed to assess the startle response of larvae to sound vibration stimuli.

### 4.9. Images Acquisition and Statistical Analysis

The results of the WISH experiment were photographed using a stereomicroscope (Olympus, MVX10, Tokyo, Japan), while the readings of phenotype in other experiments were scanned via a confocal microscopy (Nikon, A1-DUT, Tokyo, Japan). For imaging, the larvae were anesthetized with tricaine MS-222 (Sigma, #A5040) and mounted in 0.6% low-melting agarose with a lateral view. Imaris X64 software (version 9.0.1) was used to reconstruct three-dimensional images and adjust contrast. To distinguish Alexa Fluor^TM^ 555 from Alexa Fluor^TM^ 647, Alexa Fluor^TM^ 647 was painted in pseudo blue color, while the former was painted in red. GraphPad Prism (version 8.0.2) supported the whole statistical analyses. All data were repeated for more than three times and presented as mean ± standard error of the mean (SEM). An unpaired two-tailed student’s *t*-test was performed for two-group comparisons, while multiple comparisons were illustrated using one-way ANOVA. The *p* value less than 0.05 (*p* < 0.05) was considered as significantly different. *p* < 0.05, *p* < 0.01, *p* < 0.001, and *p* < 0.0001 were symbolized with “*, **, ***, and ****”, respectively, and “ns” represented no significance.

## 5. Conclusions

In this study, a temporal and spatial expression pattern of *ndrg2* was drawn via WISH and specific expression of *ndrg2* both in the HCs of the otic vesicle and the neuromasts in the pLL was detected. The morphological and functional deficiency occurred in the crista HCs and neuromasts of the *ndrg2* morphants and mutants, which could be rescued by co-injection of the exogenous *ndrg2* mRNA. Meanwhile, larvae that lacked *ndrg2* were desensitized in response to sound vibration stimuli under startle response tests. Additionally, loss of HCs in the *ndrg2* mutants accompanied by no detectable HCs apoptosis and supporting cells changes could be rescued by blocking Notch signaling, which suggested that *ndrg2* was associated with HCs’ differentiation mediated by Notch. In summary, the present study firstly demonstrated that *ndrg2* regulated HCs’ morphogenesis and auditory sensory function involved in Notch signaling pathway during the development of zebrafish. As a common vertebrate model organism, zebrafish exhibited unique advantages in hearing loss research including easy staining and imaging in vivo, not requiring complex anatomic procedures, easy genetic manipulation, suitable rapid large-scale phenotypic screening, etc. The reported functional roles of *ndrg2* in the development of auditory organs provided a pathway for discovering and identifying potential deafness genes. This work will not only deepen the understanding of *ndrg2* functions, but also give new insights into the regulation mechanism of HC development.

## Data Availability

The data presented in this study are available upon request from the corresponding author.

## Supplementary Materials

The supporting information can be downloaded at: https://www.mdpi.com/article/10.3390/ijms241210002/s1.
