# Supplementary material for: The ndrg2 Gene Regulates Hair Cell Morphogenesis and Auditory Function during Zebrafish Development
Source: Int J Mol Sci. 2023 Jun 11;24(12):10002. doi: 10.3390/ijms241210002 (PMC10297845; doi:10.3390/ijms241210002)
Supplement: Supplementary file 1 [file ijms-24-10002-s001.zip › ijms-2396349-supplementary.pdf]

Supplementary Material

Table S1

Primer information used in this study was listed as follows.

Primer name	Primer sequence (5’ - 3’)	Purpose
ndrg2-ISH-F	TTACTGCCCGGTCAATCAGA	Preparation of *ndrg2* probe for
ndrg2-ISH-R	GGAAGACGTCAGGTTGGAGA	*in situ* hybridization
ndrg2-MO-F	AAAGTGCGAGATCAAGAGCG	Efficiency validation of
ndrg2-MO-R	TTGACCAGAACCAGACCCTC	*ndrg2*-specific morpholino
ndrg2-sgRNA-F	TAATACGACTCACTATA**GCAGGAGATCGCCATCACGG**GTTTTAGAGCTAGAAATAGC	Preparation of *ndrg2* sgRNA for *ndrg2* mutants generation
ndrg2-sgRNA-R	AAAAAAAGCACCGACTCGGTGCCAC	
ndrg2-sgRNA-Seq-F	TTAGCTCAGCCAGTCGAACA	Identification of *ndrg2* mutation
ndrg2-sgRNA-Seq-R	TTTTCCAAGTGTCCAGCAGG	at target site
ndrg2-mRNA-BamHI-F	CGGGATCCAAAGTGCGAGATCAAGAGCG	Preparation of exogenous *ndrg2*
ndrg2-mRNA-XbaI-R	GCTCTAGAGTTTAAAGGGCTGGCTGGTC	mRNA for rescue experiment
eya1-ISH-F	TTTAGCACAGACGGATTCCA	Preparation of *eya1* probe for *in*
eya1-ISH-R	CTACAAATACTCCAGGTCCA	*situ* hybridization

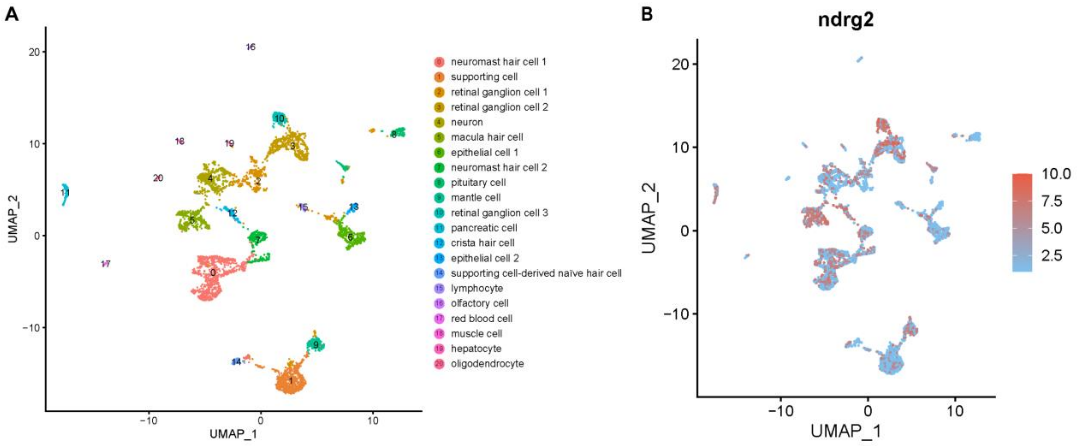

Figure S1. The *ndrg2* gene was highly enriched in clusters of crista hair cell (HC), macula HC and neuromast HC as well as in retinal ganglion cell and neuron. **(A)** Cell clusters numbered from 0 to 20 were obtained based on the analysis of HCs single-cell RNA sequencing data (GSE221471) in our previous work. **(B)** Distributions of the *ndrg2* gene in different cell cluster.

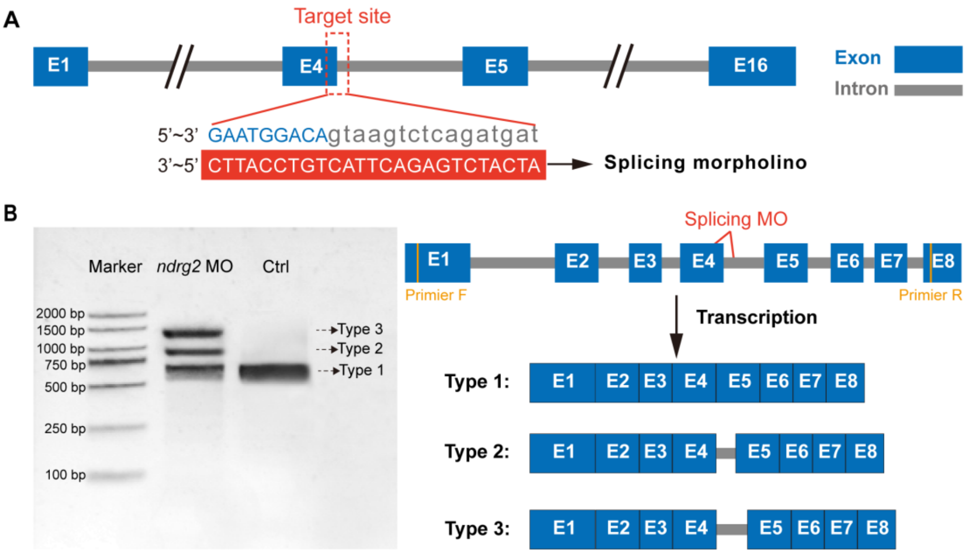

Figure S2. Effective mis-splicing at the target site was realized by microinjection of *ndrg2*-specific splicing-blocking morpholino into zebrafish embryos at one-cell stage. **(A)** Schematic diagram of knockdown of the *ndrg2* gene utilizing splicing-blocking morpholino. **(B)** The efficiency of *ndrg2*-specific splicing-blocking morpholino was verified by PCR and results of multiple electrophoretic bands suggested that several types of splicing occurred at the target site.

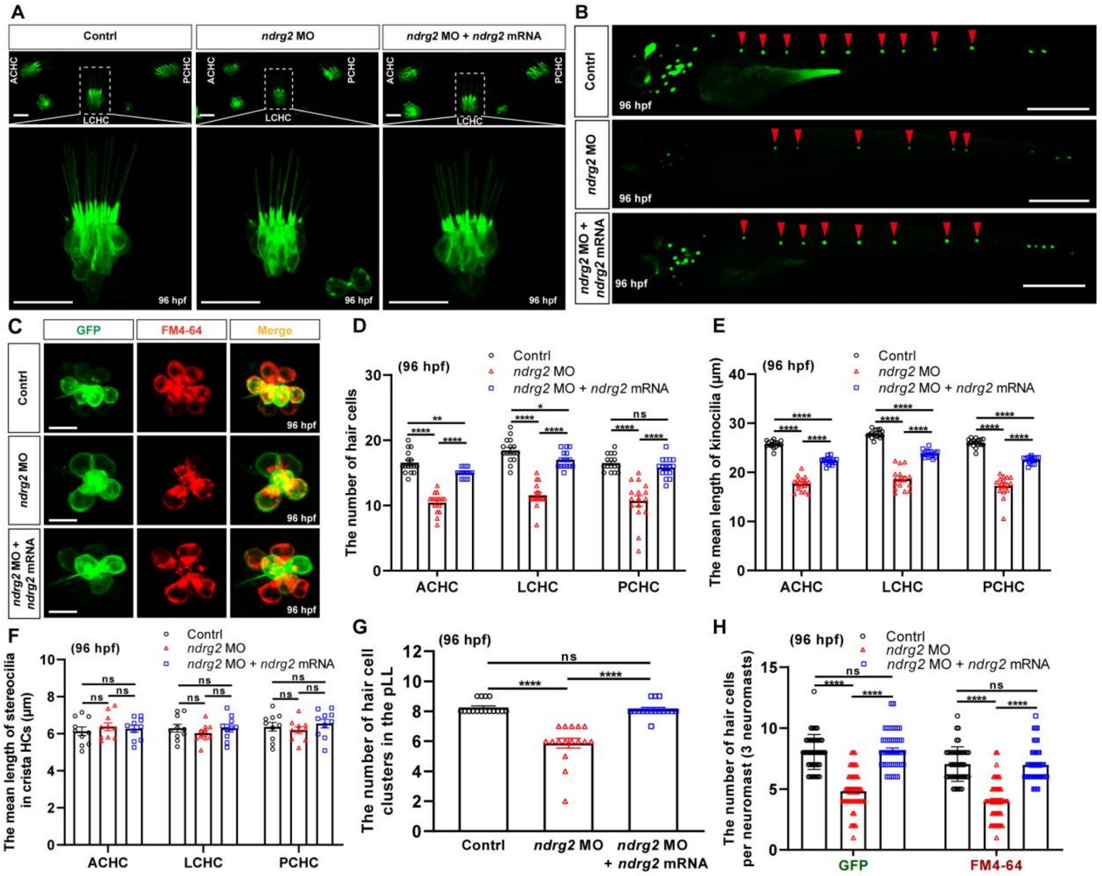

Figure S3. The defective phenotypes of HCs both in crista hair cells (HCs) and posterior lateral line (pLL) system were also detected in larvae at 96 hpf when the *ndrg2* gene was knockdown. **(A)** Representative fluorescence images of typical three clusters of crista HCs at 96 hpf in normal, *ndrg2* morphants, and *ndrg2* mRNA rescued larvae, respectively. The enlarged images of lateral crista hair cell (LCHC) (marked with white dashed box) were displayed in corresponding larvae. Scale bars: 20 µm. **(B)** Representative fluorescence graphs of HC clusters (marked with red arrowheads) in pLL at 96 hpf in the control, *ndrg2* morphants, and *ndrg2* mRNA rescued groups, respectively. Scale bars: 500 µm. **(C)** Representative enlarged micrographs of HC cluster (green color) and functional HC cluster (red color) in single neuromast of pLL at 96 hpf in control, *ndrg2* morphants, and *ndrg2* mRNA rescued groups, respectively. Scale bars: 10 µm. **(D-G)** Statistical analysis of the number of crista HCs, the mean length of kinocilia and stereocilia in crista HCs as well as the number of HC clusters in pLL at 96 hpf in the control, *ndrg2* morphants, and *ndrg2* mRNA rescued groups, respectively (d, e, g, n = 16; f, n = 10). **(H)** Statistical analysis of the number of HCs and functional HCs per neuromast at 96 hpf in control, *ndrg2* morphants, and

ndrg2 mRNA rescued groups, respectively (n = 48). Symbols of *, **, and **** above bars represent $P < 0.05$, $P < 0.01$ and $P < 0.0001$, respectively. ns, no significance.

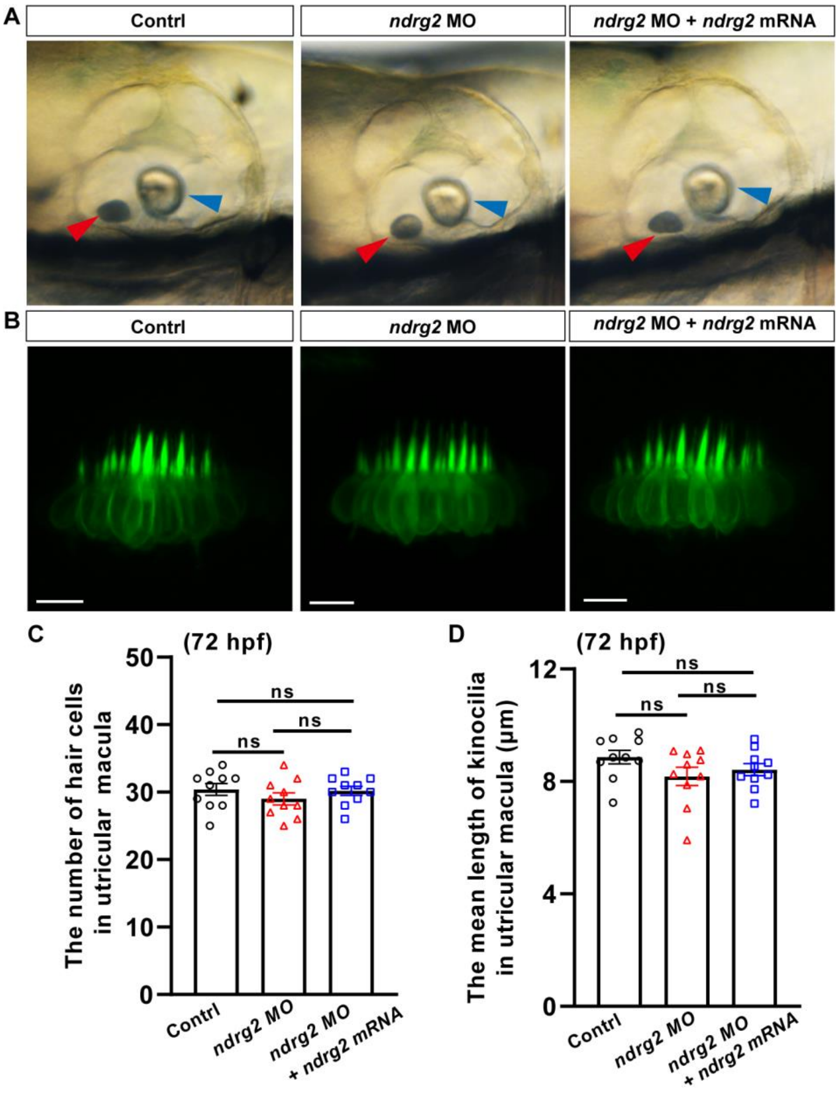

Figure S4. The distinct morphological changes were failed to observe in utricular otolith, saccular otolith and utricular macula hair cells (HCs) in otic vesicle. **(A)** The phase-contrast images of otic vesicles in normal, *ndrg2* morphants, and *ndrg2* mRNA rescued larvae at 5 dpf, respectively. The utricular otolith and saccular otolith were marked with red arrowhead and blue arrowhead, respectively. **(B)** Representative fluorescence graphs of utricular macula HCs in otic vesicle at 72 hpf in control, *ndrg2* morphants, and *ndrg2* mRNA rescued groups, respectively. Scale bars: 10 μm. (**C, D**) Statistical analysis of the number and the mean length of kinocilia in utricular macula

HCs at 72 hpf in the control, *ndrg2* morphants, and *ndrg2* mRNA rescued groups, respectively (*n* = 10). ns, no significance.

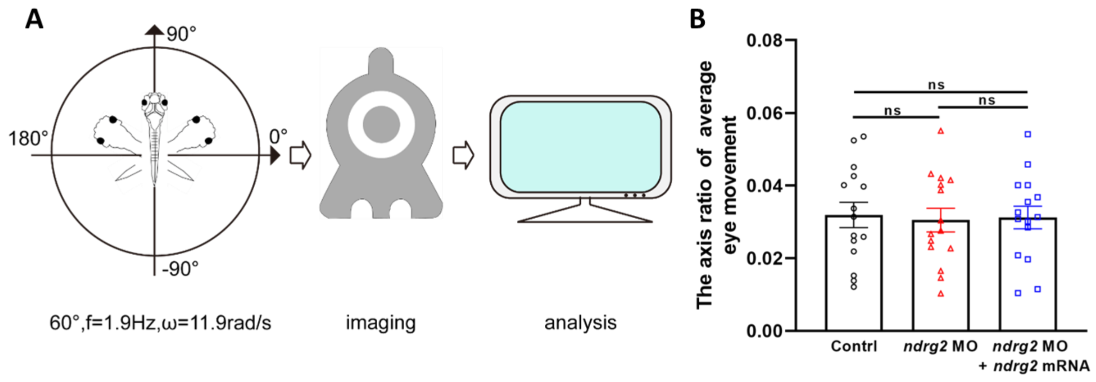

Figure S5. Vestibulo-ocular reflex (VOR) assay was used to evaluate the vestibular function and there was no significant difference of amplitude of eye movements in zebrafish larvae at 5 dpf with *ndrg2* deficiency. **(A)** The schematic diagram showed the rotatory trajectory of the larva during VOR test. **(B)** Statistical analysis the axis ratio of eye movement in normal, *ndrg2* morphants and *ndrg2* mRNA rescued larvae at 5 dpf (*n* = 15). ns, no significance.

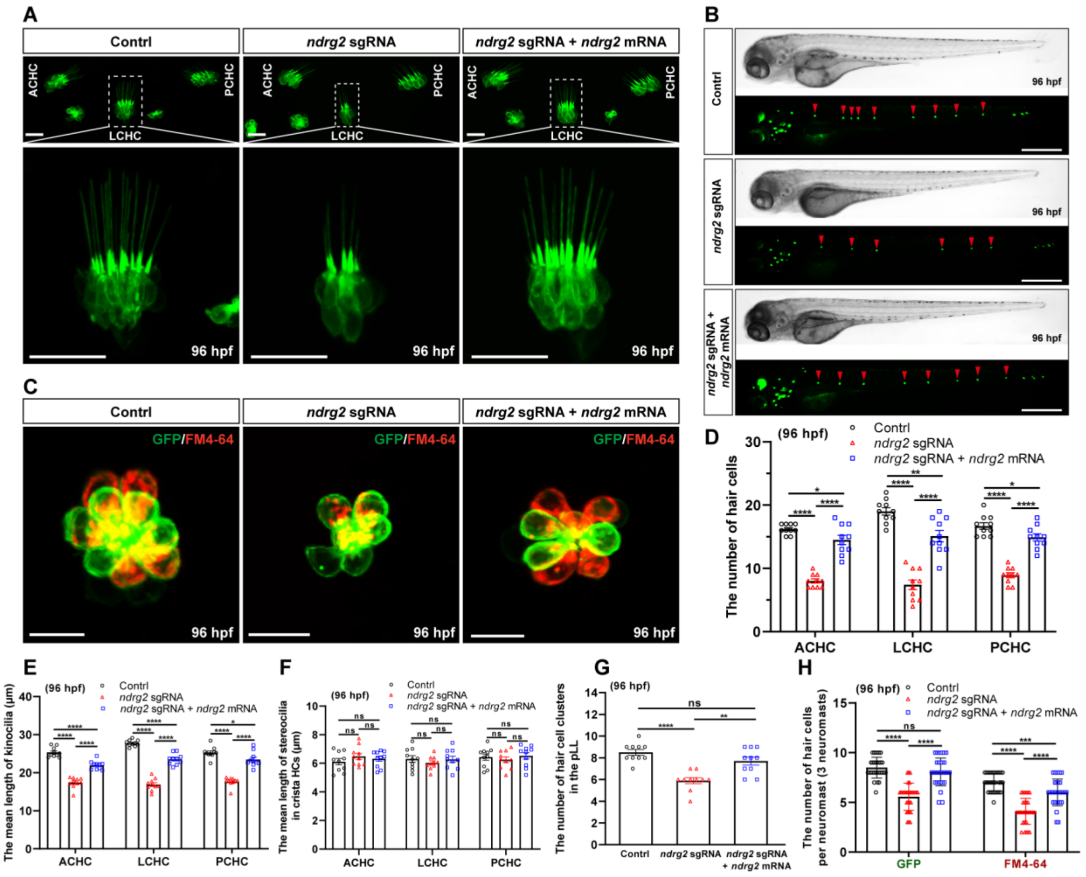

Figure S6. Knockout of the *ndrg2* gene disrupted hair cell (HC) morphogenesis in zebrafish larvae at 96 hpf. **(A)** Representative fluorescence graphs of the typical three clusters of crista HCs in normal, *ndrg2* mutants, and *ndrg2* mRNA rescued groups at 96 hpf, respectively. The enlarged images of lateral crista hair cell (LCHC) (marked with white dashed box) were shown in corresponding group. Scale bars: 20 μm. **(B)** Phase-contrast and fluorescence images of HC clusters (marked with red arrowheads) in posterior lateral line (pLL) at 96 hpf in the control, *ndrg2* mutants, and *ndrg2* mRNA rescued groups, respectively. Scale bars: 500 μm. **(C)** Overlapped fluorescence images of a representative neuromast in pLL from normal, *ndrg2* mutants, and *ndrg2* mRNA rescued larvae at 96 hpf, respectively. The green and red signals represented HCs and functional HCs, respectively. Scale bars: 10 μm. **(D-H)** Statistical analysis of the morphological changes of HCs appeared in ampulla crista and neuromasts of pLL at 96 hpf in the control, *ndrg2* mutants, and *ndrg2* mRNA rescued groups, respectively (d-g, n = 10; h, n =30). Symbols of *, **, ***, and **** above bars represent $P < 0.05$, $P < 0.01$, $P < 0.001$, and $P < 0.0001$, respectively. ns, no significance.
